# Metabolic Regulation of Two *pksCT* Gene Transcripts in *Monascus ruber* Impacts Citrinin Biosynthesis

**DOI:** 10.3390/jof9121174

**Published:** 2023-12-07

**Authors:** Yi He, Lisha Zhu, Xingxing Dong, Aoran Li, Suyin Xu, Liling Wang, Yanchun Shao

**Affiliations:** 1 National R&D Center for Se-Rich Agricultural Products Processing, Hubei Engineering Research Center for Deep Processing of Green Se-Rich Agricultural Products, School of Modern Industry for Selenium Science and Engineering, Wuhan Polytechnic University, Wuhan 430023, China; zhulisha199888@126.com (L.Z.); dongxingxing@163.com (X.D.); 2 Key Laboratory for Deep Processing of Major Grain and Oil, Ministry of Education, Hubei Key Laboratory for Processing and Transformation of Agricultural Products, School of Food Science and Engineering, Wuhan Polytechnic University, Wuhan 430023, China; liaoran618@outlook.com (A.L.); xqw1koss@163.com (S.X.); 3 College of Food Science and Engineering, Tarim University, Alar 843300, China; 120060036@taru.edu.cn; 4 College of Food Science and Technology, Huazhong Agricultural University, Wuhan 430070, China

**Keywords:** alternative splicing, citrinin, glycolysis, lipid metabolism, *Monascus ruber*, RNA interference

## Abstract

Citrinin (CIT), a secondary metabolite produced by the filamentous fungi *Monascus* species, exhibits nephrotoxic, hepatotoxic, and carcinogenic effects in mammals, remarkably restricting the utilization of *Monascus*-derived products. CIT synthesis is mediated through the *pksCT* gene and modified by multiple genetic factors. Here, the regulatory effects of two *pksCT* transcripts, *pksCT*α, and *pksCT*β, generated via pre-mRNA alternative splicing (AS), were investigated using hairpin RNA (ihpRNA) interference, and their impact on CIT biosynthesis and the underlying mechanisms were assessed through chemical biology and transcriptome analyses. The CIT yield in ihpRNA-pksCTα and ihpRNA-pksCT (α + β) transformants decreased from 7.2 μg/mL in the wild-type strain to 3.8 μg/mL and 0.08 μg/mL, respectively. Notably, several genes in the CIT biosynthetic gene cluster, specifically *mrl3*, *mrl5*, *mrr1,* and *mrr5* in the ihpRNA-pksCT (α + β) transformant, were downregulated. Transcriptome results revealed that silencing *pksCT* has a great impact on carbohydrate metabolism, amino acid metabolism, lipid metabolism, and AS events. The key enzymes in the citrate cycle (TCA cycle) and glycolysis were significantly inhibited in the transformants, leading to a decrease in the production of biosynthetic precursors, such as acetyl-coenzyme-A (acetyl-coA) and malonyl-coenzyme-A (malonyl-coA). Furthermore, the reduction of CIT has a regulatory effect on lipid metabolism via redirecting acetyl-coA from CIT biosynthesis towards lipid biosynthesis. These findings offer insights into the mechanisms underlying CIT biosynthesis and AS in *Monascus*, thus providing a foundation for future research.

## 1. Introduction

*Monascus* spp. have a rich history in China’s fermentation industry owing to their production of valuable secondary metabolites, including *Monascus* pigments and monacolin K [1,2]. Among these, *Monascus* pigments not only serve as natural edible colorants but also exhibit potent anticancer properties [3]. A notable *Monascus*-derived fermented product is red yeast rice, which is extensively used as both a functional food and traditional medicine [4]. However, certain *Monascus* species can produce citrinin (CIT), a mycotoxin with hepatotoxic effects, leading to enzyme inhibition, mitochondrial membrane potential disruption, and ultimately apoptosis, as observed in mice [5,6]. The hepatotoxicity and nephrotoxicity of CIT have raised global concerns regarding food contamination [7]. Consequently, several regions and countries, including the European Union, Japan, Korea, and China, have implemented strict limits of food and feed on CIT content [8], substantially limiting the application and development of *Monascus*-derived products. To effectively combat CIT contamination, understanding the CIT biosynthetic pathway is crucial.

Research regarding the CIT biosynthetic pathway has been ongoing since its discovery in 1930 [9]. Between the 1950s and 1990s, researchers worldwide have employed various techniques, such as radioisotope-, stable isotope-, and nuclear magnetic resonance-based methods [10,11,12]. The 7.9 kb *pksCT* gene (GenBank: AB167465.1), encoding type I nonreducing polyketide synthase, has been identified in *Monascus purpureus*, *Monascus ruber*, *Monascus aurantiacus*, and *Penicillium expansum* [13,14]. CIT biosynthetic precursors include acetyl-coA and malonyl-coA. The *pksCT* gene is responsible for forming the polyketide skeleton, which undergoes transformation through complex chemical reactions, including hydrolysis, dehydrogenation, and oxidation–reduction reactions, ultimately yielding mature CIT. Shimizu et al. [15] confirmed the involvement of *ctnA* as an activator gene in CIT biosynthesis in *M. purpureus*. Moreover, He and Cox [16] corrected the function of CitA, previously misidentified as an oxidase, through an assessment of the molecular processes involved in CIT biosynthesis via gene knockout and heterologous expression methods. Notably, CIT production is also influenced by diverse factors, including the environment, other metabolites, and exogenous additives. For example, the addition of lanthanum (III) ions during *M. purpureus* fermentation reduces CIT yield and enhances MP production [17]. He et al. [18] found that flavonoid supplementation results in a 78.13% CIT reduction through its effects on in vivo metabolism, particularly pyruvate and protein metabolism.

**Figure 1:**
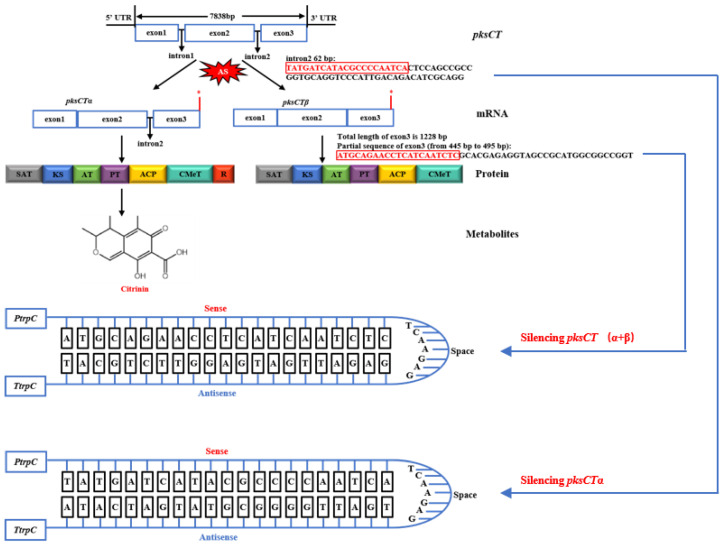
Alternative splicing (AS) of *pksCT* gene precursor mRNA and the design of hairpin structures for silencing citrinin *pksCT*α and *pksCT* (α + β) genes. SAT: starter unit acyl transferase; KS: β-ketoacyl synthase; AT: acyl transferase; ACP: acyl carrier protein; PT: product template; R: reductive release domain; CMeT: methyl transferase (* represents terminator).

Grützmann et al. [19] conducted a comprehensive study of 23 fungi species across various genera, revealing that approximately 6.4% of fungal genes possess alternative splicing (AS) capabilities. These genes play crucial roles in fungal growth, development, differentiation, pathogenicity, and secondary metabolite synthesis. Zhang and Miyake [20] reported that the methyltransferase gene (*MpLaeA*) in *Monascus pilosus* produces two alternatively spliced mRNAs, *d-MpLaeA* and *l-MpLaeA*, possibly regulating monacolin K biosynthesis. Furthermore, He and Cox [16] were the first to confirm that the *pksCT* gene can generate two transcript variants in the transcription process: *pksCT*α, containing a 62–base pair (bp) intron sequence, and *pksCT*β, lacking the same intron sequence (Figure 1). The 62 bp intron sequence is positioned between the acyl carrier protein- and reductase (R)-domain-encoding regions. Consequently, *Aspergillus oryzae* carrying *pksCT*α retain CIT-producing capabilities, whereas strains with *pksCT*β (without the 62 bp intron sequence) cannot produce CIT due to R-domain disruption. Although AS of the *pksCT* gene precursor mRNA has been discovered, its impact on CIT biosynthesis needs to be further elucidated. Recently, RNA sequencing has emerged as a powerful tool for deciphering biosynthetic pathway mechanisms and obtaining comprehensive insights [21]. For example, Yuan et al. [22] elucidated the mechanism underlying dynamic changes in fatty acid and phytosterol content during foxtail millet seed development via transcriptome analysis. Ouyang et al. [23] demonstrated CIT production reduction upon genistein addition during fermentation, which influenced transcription levels. Zorin et al. [24] identified two key genes involved in mycorrhiza-specific AS events of pea mycorrhizal roots using transcriptomics.

The aim of this study was to determine the influence of the two *pksCT* transcripts, *pksCT*α and *pksCTβ*, on CIT biosynthesis and elucidate the underlying mechanism using RNA interference, multiple chemical biology, and transcriptome analyses.

## 2. Materials and Methods

### 2.1. Strains and Culture Conditions

*Monascus ruber* M7 (CCAM 070120, preserved in Culture Collection of State Key Laboratory of Agricultural Microbiology, Huazhong Agricultural University), with the characteristics of high yield of *Monascus* pigments and CIT, was routinely grown on potato dextrose agar (PDA) medium for 3–5 days at 28 °C for DNA extraction [25]. The mixture was adjusted to a total volume of 1 L with distilled water afterwards. After cultivating for 15–20 days on PDA medium, 1 mL of spore suspension (adjusted to 10^5^ spores/mL with sterilized water) of M7 was inoculated into 100 mL of yeast extract sucrose (YES) medium (16 g/L of sucrose and 4 g/L of yeast extract) to stimulate CIT production. The mixture was then incubated under static conditions for 15 days of fermentation at 28 °C. *Escherichia coli* (*E. coli*) DH5α cells were cultivated in Luria–Bertani (LB) medium, which consisted of 5 g/L yeast extract, 10 g/L tryptone, and 10 g/L NaCl. For solid LB medium, 20 g/L of agar was added. The cells were incubated at 37 °C for 12–24 h. *Agrobacterium tumefaciens* EHA105 cells were cultivated in LB medium or on solid LB medium at 28 °C for 36–72 h. The competent cells were obtained from AngYu Biotechnologies Co., Ltd. (Shanghai, China).

### 2.2. Construction of the Fungal Expression Vector pC3300-neo

To select the positive *Monascus* transformants from screening plates, *neo* (neomycin resistant gene, 1221 bp) as a selection marker was connected to the expression vector pC3300. Briefly, *neo* with restriction sites was amplified by polymerase chain reaction (PCR) (Bio-rad T100, Hercules, CA, USA) from the pKN1 plasmid using specific primers (Table 1). The PCR products of the *neo* and pC3300 plasmids were double-digested with QuickCut *Xho* I and QuickCut *Eco*R I and linked together using T4 DNA ligase after purification. Upon double digestion with QuickCut *Xho* I and QuickCut *Eco*R I, the recombinant plasmid derived from the positive *E. coli* transformant exhibited two distinct DNA bands in lane 1 (Figure S1a). The larger band matched the size of the pC3300 plasmid (>5000 bp), while the smaller band, located between 750 bp and 1500 bp, corresponded to the PCR product of *neo* (lane 2). This confirmed the presence of the *neo* gene (1221 bp) in the recombinant plasmid. ApexHFHS DNA Polymerase FL was obtained from Accurate Biotechnology (Hunan) Co., Ltd. (Shanghai, China). QuickCut restriction enzymes and T4 DNA ligase were purchased from Takara Biomedical Technology (Beijing) Co., Ltd. (for detailed construction, see Supplementary Methods).

### 2.3. Plasmid Construction for Silencing pksCTα and pksCT (α + β) Genes

To interfere with the expression of specific genes, the method of constructing hairpin RNA was used in this study [26]. As shown in Figure 1, the intron 2 fragment of the *pksCT*α gene consists of 62 bp, the first 21 bp at the 5′ end were selected as a trigger for forming ihpRNA structure to silence the *pksCT*α gene. In addition, the 21 bp region (from 445 bp to 466 bp) of exon 3 was chosen as the trigger for forming ihpRNA structure to silence the *pksCT* (α + β) gene. The primers used in plasmid construction are listed in Table 1. Then these recombinant plasmids were confirmed using PCR and double digestion (Figure S1c–e) (for detailed construction and confirmation, see Supplementary Methods).

### 2.4. Agrobacterium Tumefaciens-Mediated Transformation (ATMT)

The recombinant plasmids were first transformed into *Agrobacterium tumefaciens* EHA105 competent cells, and then transformed into *Monascus* using the ATMT method as described by Shao et al. [25]. Briefly, the recombinant plasmids were transformed into *Agrobacterium tumefaciens* EHA105 cells and then cocultured with the spores of M7 on an induction medium for 2 days at 25 °C. After that, the cocultured products were transferred to the screening medium and then cultivated for 5 days at 28 °C. The screening medium contained 15 mg/L of G418 and 0.5 g/L of cefotaxime sodium (to inhibit the growth of *Agrobacterium tumefaciens*) to select the positive *Monascus* transformants.

### 2.5. Plasmid Integration Confirmation

Genomic DNA was extracted from *Monascus* mycelium using the CTAB method described by Shao et al. [25] after 3–5 days of cultivation on PDA medium. The plasmid integration of positive *Monascus* transformants was confirmed by PCR with primers *Hin*d III-*PtrpC* Forward and *Xba* I-*TtrpC* Reverse (Figure S1f,g, Table 1) (for detailed confirmation, see Supplementary Methods).

### 2.6. Measurement of CIT Content

CIT content was quantified by the method described by Guo et al. [27] with moderate modifications. Briefly, CIT was extracted from the fermentation broth, then quantified by an ultraperformance liquid chromatography (UPLC) (Waters, Milford, MA, USA) (for details, see Supplementary Methods).

### 2.7. Library Construction and Sequencing

The mycelia of M7, ihpRNA-*pksCT*α, and ihpRNA-*pksCT* (α + β) transformants, cultured in YES medium at 28 °C and 150 rpm for 5 d, were rapidly frozen and ground using liquid nitrogen. Total RNA was extracted from *Monascus* samples using TRIzol^®^ Reagent (Takara Biomedical Technology (Beijing) Co., Ltd., Beijing, China), and genomic DNA was removed using DNase I (Takara Biomedical Technology (Beijing) Co., Ltd., Beijing, China). RNA degradation and contamination were evaluated on 1% agarose gels. Then RNA quality was measured by a 2100 Bioanalyser (Agilent Technologies Co. Ltd., Santa Clara, CA, USA) and quantified using a NanoDrop 2000 spectrophotometer (Thermo Fisher Scientific Inc., Wilmington, NC, USA). The high-quality RNA samples (OD260/280 = 1.8~2.2, OD260/230 ≥ 2.0, RIN ≥ 8.0, 28S: 18S ≥ 1.0) were used to construct a sequencing library. RNA purification, reverse transcription, library construction, and sequencing were performed at Shanghai Majorbio Bio-Pharm Biotechnology Co., Ltd. (Shanghai, China).

### 2.8. Quality Control and Read Mapping

The raw paired-end reads were trimmed and quality controlled by Fastp (https://github.com/OpenGene/fastp (accessed on 1 July 2023)), and the clean reads were separately aligned to the reference genome with orientation mode using HISAT2 software v.2.2.1.0 (http://ccb.jhu.edu/software/hisat2/index.shtml (accessed on 24 July 2020)). Moreover, the mapped reads of each sample were assembled by StringTie (https://ccb.jhu.edu/software/stringtie (accessed on 12 March 2021)) [28] and then compared with the reference transcripts using Gffcompare (http://ccb.jhu.edu/software/stringtie/gffcompare (accessed on 23 July 2021)) to identify new transcripts.

### 2.9. Differentially Expressed Genes (DEGs) and Functional Enrichment

The expression level of each gene was calculated using the transcripts per million reads (TPM) method to identify DEGs between two different samples [29]. To ensure accuracy, genes showing *p* < 0.05 and fold change (FD) > 2 were considered the standard for significantly different expressions. Serial analysis of gene expression (RSEM) was used to quantify gene abundances (http://deweylab.biostat.wisc.edu/rsem (accessed on 15 February 2020)). In addition, functional annotation and enrichment analysis, including annotated to Gene Ontology (GO, http://www.geneontology (accessed on 1 January 2023)) database and Kyoto Encyclopedia of Genes and Genomes (KEGG, http://www.genome.jp/kegg (accessed on 1 January 2023)) database, were performed to identify which DEGs were significantly enriched in GO terms and metabolic pathways at *p* ≤ 0.05 compared with the whole transcriptome background. GO functional enrichment and KEGG pathway analysis were carried out by Goatools (https://github.com/tanghaibao/Goatools (accessed on 3 February 2023)) and KOBAS (http://kobas.cbi.pku.edu.cn/home.do (accessed on 19 February 2017)), respectively.

### 2.10. Reverse Transcription Quantitative PCR (RT-qPCR) Analysis of Genes Involved in CIT Biosynthesis

To validate the accuracy of the transcriptome data, we quantified the key genes involved in CIT biosynthesis using the RT-qPCR method described by Ouyang et al. [23] (for details, see Supplementary Methods).

### 2.11. Analysis of Alternative Splicing

All the alternative splicing events that occurred in our sample were identified using the program rMATS [30]. The splicing differences were mainly detected as skipped exon (SE), alternative 5′/3′ splice site (A5SS/A3SS), mutually exclusive exon (MXE), and retained intron (RI) [31].

### 2.12. Statistical Analysis

The data were expressed as mean ± standard deviation, and all experiments were performed in triplicate in this study. Statistical analysis was carried out by one-way analysis of variance (ANOVA) using the IBM SPSS Statistics of version 19.0 software (IBM Corporation, Almonk, NY, USA). *p* ˂ 0.05 and *p* ˂ 0.01 were considered statistically significant and extreme significant, respectively.

## 3. Results

### 3.1. CIT Content Analysis

It has been proven that pksCT plays a vital role in regulating CIT biosynthesis. Thus, we created strains that silenced two transcripts (Figure S1f,g), pksCTα and pksCTβ, to investigate the impact of pksCT on CIT biosynthesis and other secondary metabolisms. After being subcultured for three generations on the screening medium containing 15 mg/L of G418, a total of six transformants were obtained. Out of these, three transformants were silenced with pksCTα, while the remaining transformants were silenced with pksCT (α + β). The ihpRNA-*pksCT*α transformant and ihpRNA-*pksCT* (α + β) transformant were selected for further study due to their lower CIT production compared to the other transformants.

**Figure 2:**
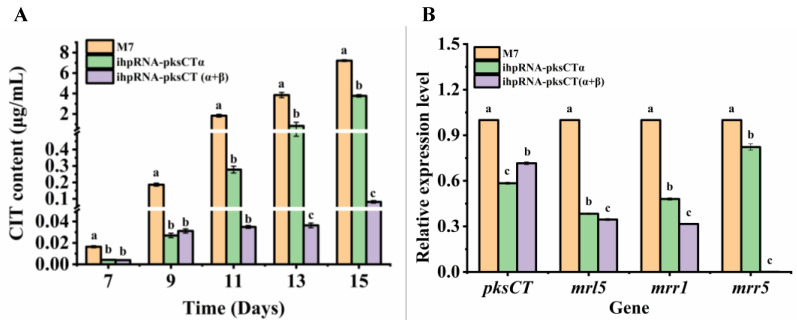
Citrinin (CIT) yield of three different *Monascus* strains during a 15-day fermentation (**A**) and RT-qPCR verification of the gene expression level in CIT biosynthesis (**B**). Different letters indicate statistical differences (*p* < 0.05) between strains. M7: the wild-type strain; ihpRNA-pksCTα: the transformant strain with silenced *pksCTα* gene; ihpRNA-pksCT (α + β): the transformant strain with silenced *pksCT*(α + β) gene.

Figure 2A shows the changes in CIT content during a 15-day fermentation period for the parental strain M7 and the positive *Monascus* transformants. CIT production in M7 substantially increased to 0.186 μg/mL on the ninth day, being nearly 11-fold higher than that on the seventh day. Similarly, the ihpRNA-*pksCT*α transformant exhibited a significant increase in CIT production, increasing from 0.004 μg/mL on the seventh day of fermentation to 0.027 μg/mL on the ninth day. The ihpRNA-*pksCT* (α + β) transformant also experienced a marked increase in CIT production, reaching 0.031 μg/mL from an initial level of 0.004 μg/mL during a 7-day fermentation period. No significant differences (*p* > 0.05) in CIT production were observed between the ihpRNA-*pksCT*α transformant and the ihpRNA-*pksCT* (α + β) transformant on either day seven or day nine.

Comparatively, on the ninth day of fermentation, CIT content was reduced by more than sixfold in the ihpRNA-*pksCT*α transformant and the ihpRNA-*pksCT* (α + β) transformant compared with the parental strain M7. Furthermore, as culture time was extended, CIT content in M7 accumulated to 7.216 μg/mL on the fifteenth day of fermentation, surpassing that of the ihpRNA-*pksCT*α transformant and the ihpRNA-*pksCT* (α + β) transformant by 0.91-fold and 87.3-fold, respectively. A significant difference (*p* < 0.05) was observed in CIT production between the ihpRNA-*pksCT*α transformant and the ihpRNA-*pksCT* (α + β) transformant as fermentation progressed from day 13 to day 15.

### 3.2. RNA Sequencing and Transcript Assembling

Overall, nine RNA samples from three replications were obtained from M7, ihpRNA-*pksCT*α, and ihpRNA-*pksCT* (α + β) transformants and sequenced using an Illumina NovaSeq 6000 sequencer. Each sample produced an average of 45.08 ± 0.30 million clean reads and 6.59 ± 0.04 billion clean bases. The mean values for Q20 and Q30 exceeded 98% and 94%, respectively (Supplementary Table S2). Furthermore, up to 93% of the clean reads uniquely mapped to the *M. purpureus* reference genome (GenBank assembly accession: GCA_006542485.1) (Supplementary Table S3). These results confirmed that the obtained clean reads met the requirements for transcriptomic analysis. Figure S2A,B show the results of transcript assembly, including length distribution, the relationship between transcripts and genes or exons, and potentially novel transcripts.

### 3.3. Gene Expression Analysis

**Figure 3:**
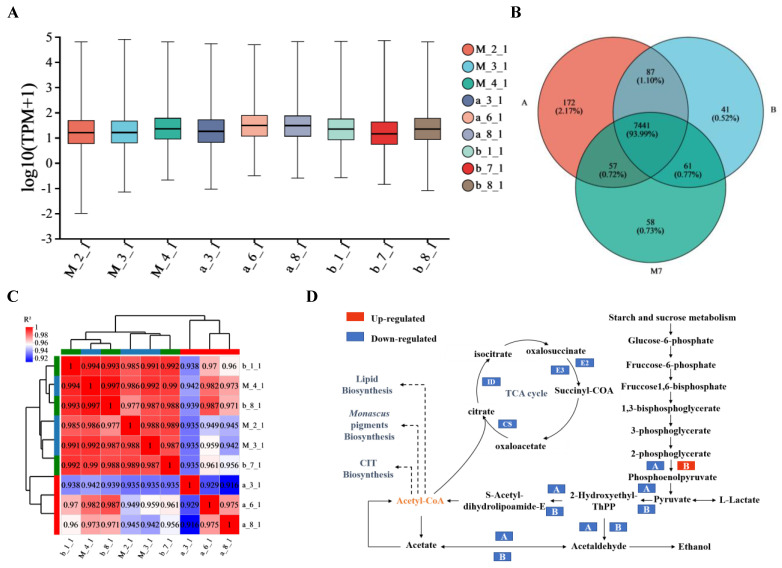
Comparative gene expression between M7 (the control group, wild-type strain), A (*Monascus* transformant with silenced *pksCT*α gene, the experimental group), and B (*Monascus* transformant with silenced *pksCT* (α + β) gene, the experimental group). (**A**) depicts the distribution of gene expression. TPM: the transcripts per million reads. (**B**) shows the pairwise comparison of the number of gene expressions. The green ball represents M7, red ball represents group A, and blue ball represents group B. (**C**) reflects the correlation between different samples. The change in color from blue to red indicates a higher correlation. (**D**) represents the change in expression levels of the differentially expressed genes in the glycolysis pathway. TCA cycle: citrate cycle.

Gene expression was calculated and normalized using RSEM ((http://deweylab.biostat.wisc.edu/rsem (accessed on 15 February 2020)), and the expression levels were reported as fragments per kilobase of transcript per million mapped reads (FPKM) [22]. Figure 3A shows the gene expression distribution across nine samples. The average gene expression for each sample fell within the moderate range (15 > FPKM ≥ 1), with an approximate FPKM value of 9. Approximately 50% of gene expression in each sample was distributed between the moderate expression level of FPKM 3 and the high expression level (60 > FPKM ≥ 15) of FPKM 47. The number of genes with high, medium, and low expression levels was similar across all samples. Moreover, a total of 7441 genes were expressed in the three strains (Figure 3B). The M7 strain shared 7498 and 7502 genes with the ihpRNA-*pksCT*α transformant and ihpRNA-*pksCT* (α + β) transformant, respectively. Additionally, 58, 172, and 41 genes were uniquely expressed in M7, ihpRNA-*pksCT*α, and ihpRNA-*pksCT* (α + β) transformants, respectively. Moreover, Spearman’s correlation coefficient analysis indicated a high correlation among biological replicates, with R^2^ values of 0.917–1 (Figure 3C), suggesting that RNAi-based targeting of the *pksCT* gene minimally influenced the expression of most genes in M7.

### 3.4. DEG Analysis

Figure S3A,B show the number of DEGs obtained from pairwise comparisons. Relative to the M7 strain, the ihpRNA-*pksCT*α transformant showed 341 upregulated and 329 downregulated DEGs, whereas the ihpRNA-*pksCT* (α + β) transformant exhibited 31 upregulated and 32 downregulated DEGs. In comparison, the ihpRNA-*pksCT* (α + β) strain showed 198 and 136 upregulated and downregulated DEGs, respectively, relative to the ihpRNA-*pksCT*α transformant. Furthermore, the first comparison group (M7_vs._A) and second comparison group (M7_vs._B) shared 26 DEGs, 644 and 37 DEGs were specific to the M7_vs._A group and M7_vs._B group, respectively. Regarding gene expression analysis for the CIT biosynthesis cluster, most genes in the cluster were downregulated. Specifically, *mrl3*, *mrl5*, *mrl6*, *mrl7*, and *mrr1* exhibited downregulation in the ihpRNA-*pksCT*α transformant, and *mrl3*, *mrl5*, *mrl6*, *mrr1*, and *mrr5* showed downregulation in the ihpRNA-*pksCT* (α + β) transformant (Table 2). Furthermore, the expression values of *pksCT* in ihpRNA-*pksCT*α and ihpRNA-*pksCT* (α + β) transformants, calculated using log_2_FC, were −0.73 and −0.43, respectively, indicating the successful inhibition of *pksCT* gene expression through hairpin interference technology.

### 3.5. Functional Annotation Analysis

**Figure 4:**
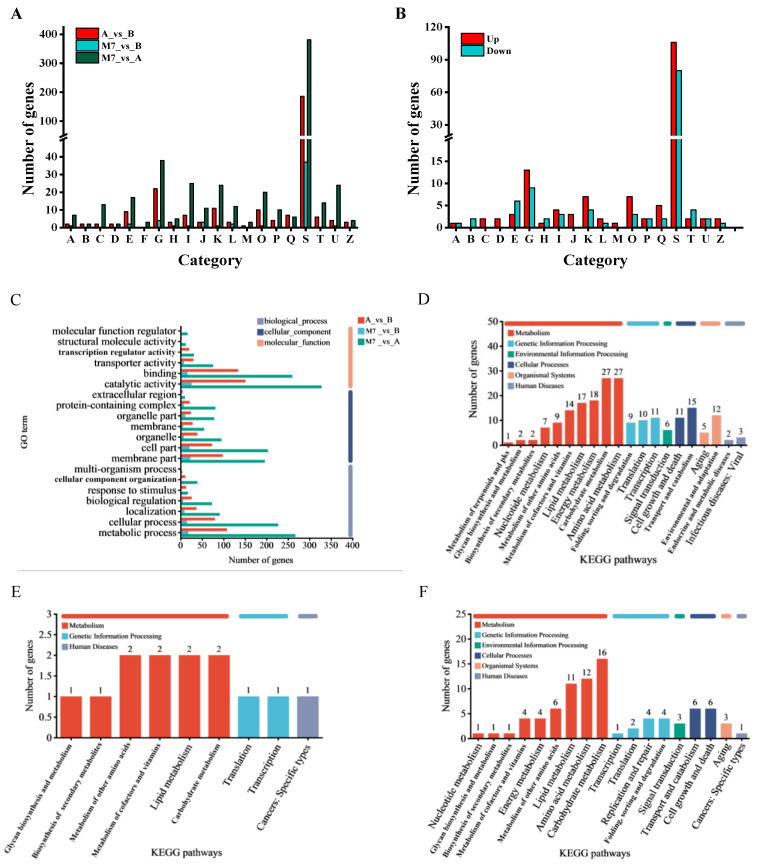
Functional annotation of the differentially expressed genes (DEGs) in three comparison groups. (**A**) shows the functional classification of DEGs after being annotated to the Cluster of Orthologous Groups database. A: RNA processing and modification; B: chromatin structure and dynamics; C: energy production and conversion; D: cell cycle control, cell division, chromosome partitioning; E: amino acid transport and metabolism; F: nucleotide transport and metabolism; G: carbohydrate transport and metabolism; H: coenzyme transport and metabolism; I: lipid transport and metabolism; J: translation, ribosomal structure, and biogenesis; K: transcription; L: replication, recombination, and repair; M: cell wall/membrane/envelope biogenesis; O: posttranslational modification; P: inorganic ion transport and metabolism; Q: secondary metabolite biosynthesis, transport, and catabolism; S: function unknown; T: signal transduction mechanisms; U: intracellular trafficking, secretion, and vesicular transport; Z: nuclear structure. (**B**) illustrates the up- and downregulated DEGs in the comparison group of A_vs._B. (**C**) is the functional classification of DEGs after being annotated to the Gene Ontology database. (**D**–**F**) depict the functional classification of DEGs in the three comparison groups of M7_vs._A, M7_vs._B, and A_vs._B, respectively, after being annotated to the Kyoto Encyclopedia of Genes and Genomes database. Group M7: the wild-type strain; group A: the transformant strain with silenced pksCTα gene; group B: the transformant strain with silenced pksCT(α + β) gene.

Functional annotation was conducted by classifying the DEGs of the three comparison groups in the Cluster of Orthologous Groups (COG) database (Figure 4A). Excluding poorly characterized functions, the most represented functional clusters in the three comparison groups included carbohydrate transport and metabolism, transcription, post-translational modification, lipid transport and metabolism, and amino acid transport and metabolism. Compared with the ihpRNA-*pksCT*α transformant, the ihpRNA-*pksCT* (α + β) transformant showed upregulation of 13 DEGs and downregulation of 9 DEGs related to carbohydrate transport and metabolism. Additionally, seven and four DEGs related to transcription were upregulated and downregulated, respectively. Furthermore, seven and three DEGs associated with post-translational modification, protein turnover, and chaperones were upregulated and downregulated, respectively (Figure 4B).

DEGs from the three comparison groups were categorized into three major categories, molecular function (MF), biological process (BP), and cellular composition (CC), following annotation via the GO database (Figure 4C). In the MF category, over 130 genes from the first group (M7_vs._A) and the third group (A_vs._B) were assigned to the GO term “catalytic activity and binding”. The second group (M7_vs._B) contained 26 and 16 genes associated with the GO terms “catalytic activity and binding”, respectively. Regarding BP terms, 108, 18, and 267 genes from the first, second, and third comparison groups, respectively, were assigned to the GO term “metabolic processes”, with slightly more genes involved in metabolic processes than cellular processes. In the CC group, the top three enriched GO terms were “membrane part”, “cell part”, and “organelle”.

DEGs from the three comparison groups were categorized into six pathways using the KEGG database (http://www.genome.jp/kegg (accessed on 1 January 2023)). These pathways included human diseases, environmental information processing, organismal systems, genetic information processing, cellular processes, and metabolic pathways. As depicted in Figure 4D, the first two representative pathways in the first group (M7_vs._A) were amino acid metabolism and carbohydrate metabolism pathways, each associated with 27 genes. These pathways were followed by energy metabolism (18 genes) and lipid metabolism (17 genes), each falling under the category of metabolism. The second representative pathway was genetic information processing, including transcription (11 genes), translation (10 genes), and folding, sorting, and degradation (nine genes).

In the second group (M7_vs._B) (Figure 4E), more than 75% of DEGs were allocated to the metabolism category. The main pathways were carbohydrate metabolism, lipid metabolism, metabolism of cofactors and vitamins, and metabolism of amino acids. As illustrated in Figure 4F, the three most abundant pathways in the third group (A_vs._B) were carbohydrate metabolism (16 genes), amino acid metabolism (12 genes), and lipid metabolism (11 genes). DEGs related to lipid metabolism were significantly upregulated in the ihpRNA-*pksCT* (α + β) transformant. The most extensively downregulated DEGs were primarily involved in the carbohydrate metabolism pathway and amino acid metabolism (Table 3).

### 3.6. Functional Enrichment Analysis

**Figure 5:**
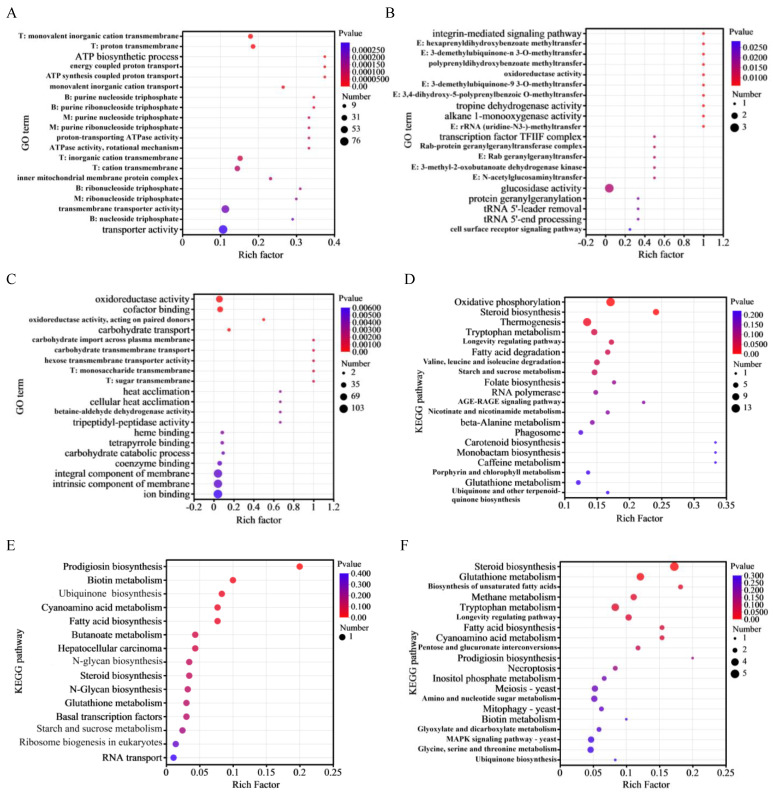
Gene Ontology (GO) database enrichment (**A**–**C**) and Kyoto Encyclopedia of Genes and Genomes (KEGG) database enrichment (**D**–**F**) analysis of the differentially expressed genes in the three comparison groups of M7_vs._A, M7_vs._B, and A_vs._B, respectively. Group M7: the wild-type strain; group A: the transformant strain with silenced pksCTα gene; group B: the transformant strain with silenced pksCT (α + β) gene. T: transporter activity; B: biosynthetic process; M: metabolic process; E: enzyme activity.

The GO enrichment analysis was used to gain insight into the biological functions of DEGs. The top 20 most significant (*p* < 0.05) GO terms from the BP, MF, and CC categories were selected. As shown in Figure 5A, 523 DEGs were classified into three GO categories in the first group (M7_vs._A): more than 70% of GO terms were BP terms, about 25% were MF terms, and the remaining terms (about 5%) were CC related. The DEGs were primarily associated with transmembrane transport of cations and protons as well as energy metabolism related to the biosynthesis and transport of ATP and purine nucleoside triphosphate.

In the second group (M7_vs._B), a large proportion of DEGs were associated with MF terms, followed by BP and CC terms (Figure 5B). The most highly enriched GO terms were related to enzyme activity, specifically the activity of methyltransferase. Interestingly, methyltransferase gene expression levels in ihpRNA-*pksCT*α and ihpRNA-*pksCT* (α + β) transformants were significantly downregulated compared with M7, with log_2_FC values of −8.54 and −8.36, respectively.

Compared with the ihpRNA-*pksCT*α transformant, the DEGs of the ihpRNA-*pksCT* (α + β) transformant were mainly associated with BP and MF terms (Figure 5C). The most highly enriched GO terms were related to oxidoreductase activity and cofactor binding as well as carbohydrate transport.

KEGG enrichment analysis was used to determine the metabolic pathways significantly associated (*p* < 0.05) with DEGs. In the first group (M7_vs._A), 251 DEGs were assigned to six main categories with 20 subcategories (Figure 5D). The two most significantly enriched pathways were oxidative phosphorylation and steroid biosynthesis. In the second group (M7_vs._B), the five most significantly enriched pathways were prodigiosin biosynthesis, biotin metabolism, ubiquinone and other terpenoid–quinone biosynthesis, cyanoamino acid metabolism, and fatty acid biosynthesis (Figure 5E). In the third group (A_vs._B), the five most significantly enriched pathways were steroid biosynthesis, glutathione metabolism, biosynthesis of unsaturated fatty acids, methane metabolism, and tryptophan metabolism (Figure 5F). Further DEG analysis in the third group (A_vs._B) revealed that genes involved in steroid biosynthesis and unsaturated fatty acid biosynthesis were upregulated in the ihpRNA-*pksCT* (α + β) transformant. Certain genes related to amino acid metabolism, such as glutathione, tryptophan, glycine, serine, and threonine metabolism genes, were downregulated in the ihpRNA-*pksCT* (α + β) transformant (Figure S4A,B).

### 3.7. Analysis of AS

**Figure 6:**
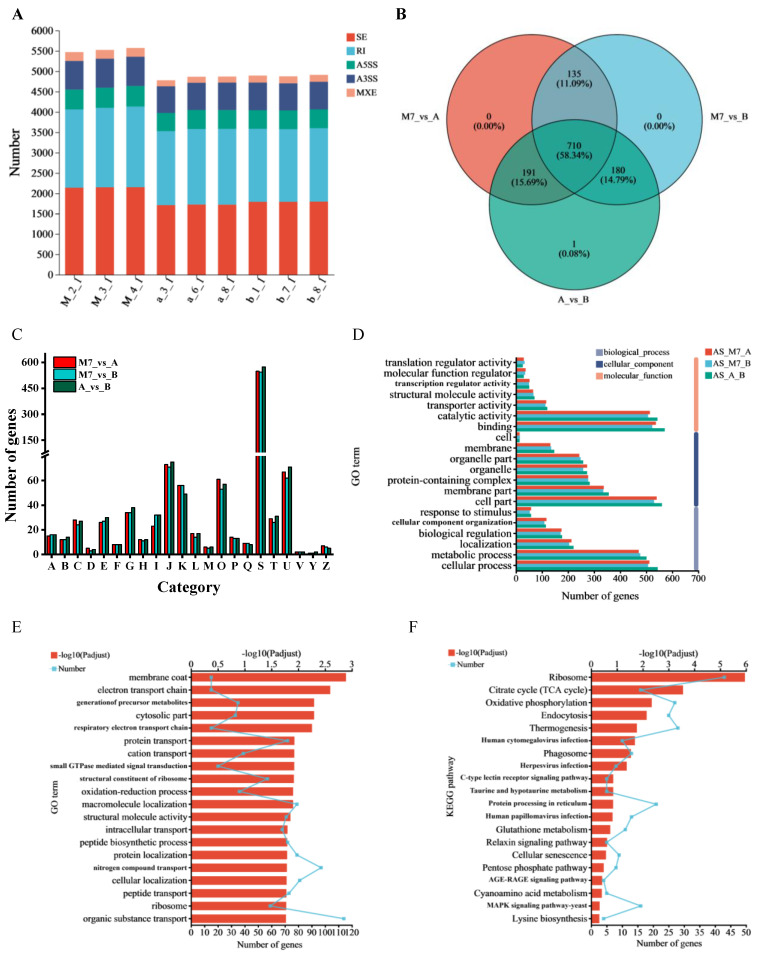
Analysis of alternative splicing (AS) in M7 and transformants. (**A**) Distribution of five AS events in different samples. (**B**) Venn analysis of AS-related genes in the three comparison groups of M7_vs._A, M7_vs._B, and A_vs._B. (**C**) Functional classification of AS-related genes after being annotated to the Cluster of Orthologous Groups database. A: RNA processing and modification; B: chromatin structure and dynamics; C: energy production and conversion; D: cell cycle control, cell division, chromosome partitioning; E: amino acid transport and metabolism; F: nucleotide transport and metabolism; G: carbohydrate transport and metabolism; H: coenzyme transport and metabolism; I: lipid transport and metabolism; J: translation, ribosomal structure, and biogenesis; K: transcription; L: replication, recombination, and repair; M: cell wall/membrane/envelope biogenesis; O: posttranslational modification; P: inorganic ion transport and metabolism; Q: secondary metabolites biosynthesis, transport, and catabolism; S: function unknown; T: signal transduction mechanisms; U: intracellular trafficking, secretion, and vesicular transport; V: defense mechanisms; Y: nuclear structure; Z: nuclear structure. (**D**) Functional classification of AS-related genes after being annotated to the Gene Ontology (GO) database. (**E**) GO enrichment analysis of AS-related genes. (**F**) Kyoto Encyclopedia of Genes and Genomes (KEGG) database enrichment analysis of AS-related genes.

In total, 5500 and 4500 AS events were identified in M7 and the transformant, respectively (Figure 6A). SE and RI events were the two most frequent AS events, followed by A3SS, A5SS, and MXE events. The AS-related genes in the three comparison groups were analyzed, with a Venn diagram showing that these groups shared 710 AS-related genes, accounting for 58.34% of all AS-related genes (Figure 6B). COG functional classification indicated that the majority of AS-related genes were involved in translation, ribosomal structure and biogenesis, intracellular trafficking, secretion and vesicular transport, post-translational modification, protein turnover and chaperones, and transcription (Figure 6C). GO annotation analysis revealed that the majority of AS-related genes were associated with the GO terms “cellular process”, “metabolic process”, “binding”, “catalytic activity”, and “cell part” (Figure 6D). The top three KEGG pathways linked to AS-related genes in the three comparison groups were translation, carbohydrate metabolism, and transport and catabolism (Figure S5). The effects of silencing the *pksCT*α and *pksCT* (α + β) genes on M7 were explored by functionally analyzing the AS-related genes in comparison group A_vs._B using the GO databases (http://www.geneontology (accessed on 1 January 2023)) and KEGG databases (http://www.genome.jp/kegg (accessed on 1 January 2023)). The five most significantly enriched GO terms (P_adjust_ < 0.01) were “membrane coat”, “electron transport chain”, “generation of precursor metabolites and energy”, “cytosolic part”, and “respiratory electron transport chain” (Figure 6E). The three most significantly enriched KEGG pathways (P_adjust_ < 0.01) were the ribosome, citrate cycle (TCA cycle), and oxidative phosphorylation pathways (Figure 6F).

## 4. Discussion

Disruption of the *pksCT* gene, responsible for encoding the CIT polyketide synthase in *Monascus*, is known to eliminate CIT production [13,32]. This highlights the importance of the *pksCT* gene in CIT biosynthesis. In the present study, RNA interference targeting this gene significantly reduced CIT yield, although silencing the *pksCT*α gene and *pksCT*(α + β) gene had differing impacts on CIT biosynthesis. The CIT yield of the ihpRNA-*pksCT*α transformant was approximately halved compared with M7, whereas the yield of the ihpRNA-*pksCT* (α + β) transformant approached zero after 15 days of fermentation. Therefore, transcriptome analysis was conducted to investigate the effects of the two *pksCT* gene transcripts on CIT biosynthesis as well as the underlying mechanisms.

The study results indicated that, in addition to reduced *pksCT* gene expression, the expression levels of other genes in the CIT biosynthetic gene cluster also decreased in both transformants. qRT-PCR results show that the expression levels of *pksCT*, *mrl5*, *mrr1*, and *mrr5* were significantly decreased in the two transformants (Figure 2B). Additionally, expression of *mrr5* in the ihpRNA-*pksCT* (α + β) transformant approached zero, consistent with the transcriptome data. Although *mrl3, mrl4*, *mrl5*, *mrl6*, *mrl7*, *mrr1*, and *mrr5* were downregulated in the ihpRNA-*pksCT*α and ihpRNA-*pksCT* (α + β) transformants, the expression levels of *mrl3*, *mrl5*, *mrr1*, and *mrr5* were lower in the latter transformant, potentially are closely associated with lower CIT yield. Huang et al. [8] found that several genes involved in CIT biosynthesis were substantially downregulated in *Monascus* strains with low CIT yield (0.02 ± 0.01 mg/L). These genes included *CtnG* (encoding carbonic anhydrase; *mrr5*), *CtnC* (encoding membrane transporter/major facilitator superfamily protein; *mrr1*), *CitB* (encoding oxygenase; *mrl2*), *CitD* (encoding dehydrogenase; *mrl4*), *CitE* (encoding dehydrogenase; *mrl6*), and *CitC* (encoding oxidoreductase; *mrl7*). The *mrr5* gene, also known as *ctnG*, functions in the formation of malonyl-coA, a precursor in CIT biosynthesis, by supplying bicarbonate. Li et al. [33] found that disrupting *ctnG* led to a significant decrease in CIT yield, reducing it by approximately 50%. This suggests that *ctnG* disruption diminishes bicarbonate and malonyl-coA levels. Therefore, compared to silencing *pksCT*α, disrupting *pksCT* (α + β) resulted in a reduction in CIT yield, which lowers the demand for biosynthesis precursors and subsequently downregulates the expression of the *mrr5* gene. Moreover, *mrr1* was downregulated in the transformants, with log_2_FC values of -1.05 and -1.59 for the ihpRNA-*pksCT*α and ihpRNA-*pksCT* (α + β) transformants, respectively. As a transporter protein, mrr1 is responsible for transporting CIT [16]. With the decrease of CIT, the expression level of *mrr1* was subsequently downregulated. Mrl3, a positive transcriptional regulatory factor, has been proven to be vital for CIT biosynthesis and functions as a pathway-specific regulator [34]. The log_2_FC values of *mrl3* in the ihpRNA-*pksCT*α and ihpRNA-*pksCT* (α + β) transformants were −0.49 and −0.57, respectively. CIT concentrations may have regulatory effects on transcriptional regulators or regulatory proteins, which downregulate the expression level of *mrl3*. Thus, the majority of genes involved in CIT biosynthesis were downregulated under the regulation of mrl3. Xie et al. [35] reported that pigR, a positive transcriptional regulatory factor in *Monascus* pigments biosynthesis, mainly governs MP biosynthesis. The transformant lacking the *pigR* gene was unable to produce pigment. Transcriptome data revealed that interfering with *pksCT*α or *pksCT* (α + β) had minimal influence on *pigR* expression, with log_2_FC values of −0.11 and 0.01 for the two transformants, respectively (Table 2). Consequently, the yield of transformants in *Monascus* pigments exhibited no significant change compared with M7.

The DEGs in the transformants were primarily associated with catalytic and metabolic processes, specifically carbohydrate metabolism, amino acid metabolism, lipid metabolism, and energy metabolism (Figure S4). Regarding carbohydrate metabolism, significant changes in DEG expression levels were associated with the glycolysis/gluconeogenesis pathway in both transformants. These changes may be responsible for the lower yield of acetyl-coA (Figure 3D). Li et al. [36] found that ctnF, an enzyme involved in CIT synthesis, exerted regulatory control over glycolytic flux, subsequently impacting CIT yield. Disruption of the *pksCT*α gene was found to hinder the transformation of glycerate-2P to phosphoenol-pyruvate, resulting in reduced enolase activity compared with M7. Conversely, silencing *pksCT* (α + β) was found to facilitate this transformation by increasing enolase activity, in contrast to the ihpRNA-*pksCT*α transformant. However, DEGs related to the transformation of pyruvate to acetyl-coA were downregulated in both transformants. Regarding amino acid metabolism, most relevant DEGs were downregulated in the ihpRNA-*pksCT* (α + β) transformant, indicating reduced protein synthesis when CIT biosynthesis capacity was almost abolished [8]. Moreover, genes involved in the degradation of branched-chain amino acids (valine, leucine, and isoleucine) were upregulated. The degradation of these amino acids likely contributed to the generation of acetyl-coA and malonyl-coA [23], suggesting that the upregulation of valine, leucine, and isoleucine serves as a compensatory mechanism to counteract the reduction in acetyl-coA and malonyl-coA levels in the ihpRNA-*pksCT* (α + β) transformant. Conversely, certain DEGs associated with amino acid metabolism were upregulated in the ihpRNA-*pksCT*α transformant, in addition to some DEGs involved in the metabolism of cofactors and vitamins. This suggests that interference with *pksCT*α may have contributed to the production of other metabolites, such as vitamins, potentially necessitating higher levels of amino acids (Figure S6). Interestingly, all DEGs related to lipid metabolism were upregulated in the ihpRNA-*pksCT* (α + β) transformant (Table 3). This indicates that a larger proportion of acetyl-coA was directed toward lipid metabolism in the ihpRNA-*pksCT* (α + β) transformant compared with the ihpRNA-*pksCT*α transformant. Duan et al. [37] reported a 51.2% increase in the yield of *Monascus* azaphilones due to reducing malonyl-coA consumption through the suppression of lipid metabolism. As shown in Figure 3D, acetyl-coA is the initial substrate for lipid biosynthesis, *Monascus* pigments or *Monascus* azaphilones biosynthesis, and CIT biosynthesis. As CIT biosynthesis was repressed, acetyl-coA will be directed towards lipid biosynthesis to enhance lipid metabolism. Regarding energy metabolism, most DEGs were downregulated in both transformants, specifically in the oxidative phosphorylation pathway and TCA cycle. As shown in Figure 3D, the key genes, such as citrate synthase (CS), isocitrate dehydrogenase (ID), dihydrolipoamide succinyltransferase E2 component, and dihydrolipoamide dehydrogenase E3 component, involved in the TCA cycle were downregulated. The suppression of the TCA cycle would lead to a decreasing production of energy, which indicates less energy was demanded in the two types of transformants. Previous studies have highlighted the pivotal role of acetyl-coA as a key biosynthetic precursor in various cellular processes and metabolism [38]. Overall, silencing two transcripts significantly decreased CIT production, which may have a regulatory effect on certain metabolic proteins, including those involved in carbohydrate metabolism, amino acid metabolism, and lipid metabolism. As a secondary metabolite, CIT is not essential for the growth and reproduction of *Monascus*. When the M7 strain reduces the burden of producing CIT, less energy, protein, and biosynthetic precursors will be needed. Therefore, in carbohydrate metabolism, the TCA cycle and glycolysis of the transformants were significantly repressed, which led to a decrease in the production of biosynthetic precursors and energy. This may further exert influence on the biosynthesis of metabolites. For example, compared with the ihpRNA-*pksCT*α transformant, the ihpRNA-*pksCT* (α + β) transformant yielded less CIT and enhanced lipid metabolism. One of the reasons is that acetyl-CoA was redirected from CIT biosynthesis into lipid metabolism. The regulatory effect on other metabolisms may also be due to the accidental effects of interfering RNA on the expression of other genes, so further research is still needed.

AS is a common post-transcriptional regulatory mechanism in fungi. He and Cox [16] reported that in the mixed transcripts of the *pksCT* gene, the majority consisted of spliced transcripts (*pksCT*β), with un-spliced transcripts (*pksCT*α) representing a minority population. In the present study, the difference in CIT yield between the two types of transformants may be attributed to the disparity in transcript abundance. Theoretically, the influence of interfering *pksCT*α and *pksCT* (α + β) on CIT yield should be similar because the spliced transcript (*pksCT*β) is not mainly responsible for the production of CIT [16], yet its high abundance and specific regulatory effects may indirectly affect CIT synthesis. The abundance of two alternatively spliced mRNAs (*d-MpLaeA* and *l-MpLaeA*) of methyltransferase gene (*MpLaeA*) was regulated by the fungal growth stages and the nutrients of medium. The *l-MpLaeA* mRNA is the predominant form and can be detected at all growth stages and in various carbon or nitrogen culture medium. The *d-MpLaeA* mRNA is an ineffectively spliced mRNA, but low levels of *d-MpLaeA* mRNA could increase the secondary metabolism of *Monascus*. Silencing *MpLaeA* by transforming antisense *d-MpLaeA* cDNA into *Monascus* led to lower production of monacolin K [20]. Similarly, the alternatively spliced *pksCT*β mRNA may favor the biosynthesis of CIT. In the ihpRNA-*pksCT* (α + β) transformant, the *pksCT*β mRNA was degraded due to the formation of the ihpRNA structure. The degradation of *pksCT*β mRNA may result in a lower CIT yield compared to when only the *pksCT*α mRNA was silenced. Additionally, the total number of AS events in the two transformants was lower than that in M7, indicating that interfering with *pksCT*α or *pksCT* (α + β) reduced post-transcriptional regulation. AS events in fungi play a role in growth, response to environmental stress, and pathogenicity [39]. For example, in *S. bambusicola*, AS events regulate biological processes, including pigment synthesis [40]. In comparison group A_vs._B, AS-related genes were primarily enriched in the ribosome, TCA cycle, and oxidative phosphorylation pathways. Most of these genes were downregulated, indicating a potential decrease in protein synthesis, energy production, and metabolic precursor synthesis in the ihpRNA-*pksCT* (α + β) transformant.

## 5. Conclusions

In conclusion, this study revealed the effects of the two *pksCT* transcripts, *pksCT*α and *pksCT* (α + β), on CIT biosynthesis as well as the underlying mechanism using RNA interference, chemical biology, and transcriptome analyses. Silencing *pksCT*α and *pksCT* (α + β) not only resulted in a significant reduction in CIT yield, but also greatly affected other metabolic and biosynthetic processes by affecting the expression levels of key genes, pyruvate transformation, TCA cycle, lipid metabolism, and AS events. This research provides insights into the regulatory mechanisms that influence CIT yield and other metabolic processes through the two *pksCT* transcripts.

## Figures and Tables

**Table 1:** List of primers used in constructing recombinant plasmids.

Target	Primer	Sequence (5′-3′)
*neo*	*Xho* I-*neo* Forward	CCGCTCGAGCCAACTCAACCCCATCG
	*Eco*R I-*neo* Reverse	CGGAATTCATCATCATGCAACATGC
*PtrpC* (α)	*Hin*d III-*PtrpC* (α) Forward	CCCAAGCTTGTCGACAGAAGATGATATTG
	*PtrpC* (α)-Reverse	CCCAATCACTCTTGATGATTGGGGCGTATGATCATACATATCGATGCTTGGGTAGAATA
*TtrpC* (α)	*TtrpC* (α) Forward	ATCATCAAGAGTGATTGGGGCGTATGATCATATTTTTCCAAGCAGCAAAGAGTGCCTTCTAG
	*Xba* I-*TtrpC* (α) Reverse	GCTCTAGAAAGAAGGATTACCTC
*PtrpC* (α + β)	*Hin*d III-*PtrpC* Forward	CCCAAGCTTGTCGACAGAAGATGATATTG
	*PtrpC* Reverse	TCAATCTCCTCTTGAGAGATTGATGAGGTTCTGCATACATATCGATGCTTGGGTAGAATA
*TtrpC* (α + β)	*TtrpC* Forward	TCTCTCAAGAGGAGATTGATGAGGTTCTGCATTTTTCCAAGCAGCAAAGAGTGCCTTCTAG
	*Xba* I-*TtrpC* Reverse	GCTCTAGAAAGAAGGATTACCTC

Red letters: restriction enzyme site; underline: hairpin structure design for interference of citrinin *pksCT* α and *pksCT* (α + β) gene.

**Table 2:** The expression changes of the main genes involved in citrinin biosynthesis and *Monascus* pigments biosynthesis in *Monascus* transformants compared with M7. (M7: the wild-type strain; ihpRNA-pksCTα: the transformant strain with silenced *pksCTα* gene; ihpRNA-pksCT (α + β): the transformant strain with silenced *pksCT* (α + β) gene.)

Gene	Predict Function	M7 vs. ihpRNA-*pksCT*α			M7 vs. ihpRNA-*pksCT* (α + β)		
		Log_2_FC	Regulate	Significant (*p* < 0.05)	Log_2_FC	Regulate	Significant (*p* < 0.05)
*mrl1*	Serine hydrolase	0.09	up	no	−0.06	down	no
*mrl2*	2-oxoglutarate-dependent dioxygenase	0	-	no	0.17	up	no
*mrl3*	Citrinin biosynthesis transcriptional activator	−0.49	down	no	−0.57	down	no
*mrl4*	Aldehyde dehydrogenase	−0.58	down	no	−0.48	down	no
*mrl5*	Glyoxalase-like domain	−1.31	down	yes	−1.50	down	yes
*mrl6*	Short chain dehydrogenase	−3.59	down	yes	−3.2	down	yes
*mrl7*	Glucose methanol choline (GMC) oxidoreductase	−1.21	down	yes	−0.27	down	no
*pksCT*	Methyltransferase activity	−0.73	down	no	−0.43	down	no
*mrr1*	Major facilitator superfamily (MFS) protein	−1.05	down	no	−1.59	down	yes
*mrr2*	Histidine phosphatase	0.073	up	no	0.258	up	no
*mrr3*	Function unknown	0.08	up	no	0.13	up	no
*mrr4*	WD repeat protein	−0.43	down	no	0.11	up	no
*mrr5*	Carbonic anhydrase	−0.22	down	no	−8.95	down	no
*mrr8*	AMP-binding enzyme	−0.36	down	no	−0.19	down	no
*pigR*	pigment biosynthesis activator	−0.11	down	no	0.01	up	no

**Table 3:** Kyoto Encyclopedia of Genes and Genomes (KEGG) database analysis of the differentially expressed genes (DEGs) in the comparison group of A_vs._B. (Group A: *Monascus* transformant with silenced *pksCT*α gene, the experimental group; Group B: *Monascus* transformant with silenced *pksCT* (α + β) gene, the experimental group).

Category	Pathway	Putative Functions of DEGs	Regulation
Carbohydrate metabolism	Butanoate metabolism	3-oxoacyl-[acyl-carrier-protein] reductase (FabG)	down
	Pentose and glucuronate interconversions	L-arabinitol 4-dehydrogenase	Down (not detected in B group)
		D-arabinitol dehydrogenase 1	up
	Inositol phosphate metabolism	scyllo-inositol 2-dehydrogenase	down
		3-phytase A	up
	Galactose metabolism	D-galactonate dehydratase	up
	Starch and sucrose metabolism	Probable beta-glucosidase A	up
	Pentose phosphate pathway	Ribokinase	down
	Glyoxylate and dicarboxylate metabolism	Catalase	down
		Glycerate dehydrogenase	down
	Amino sugar and nucleotide sugar metabolism	Chitin synthase	up
		hypothetical protein	up
		N-acetylglucosamine-6-phosphate deacetylase	down
	Glycolysis/Gluconeogenesis	Enolase	up
		Pyruvate decarboxylase	down
Amino acid metabolism	Lysine degradation	L-saccharopine oxidase	down
	Glycine, serine and threonine metabolism	NAD/NADP-dependent betaine aldehyde dehydrogenase	down
		Glycerate dehydrogenase	down
	Tryptophan metabolism	Indoleamine 2,3-dioxygenase	down
		cytochrome P450 oxidoreductase	up
		Kynureninase 2	down
	Phenylalanine metabolism	Phenylacetate 2-hydroxylase	down
		Probable 4-hydroxyphenylpyruvate dioxygenase 1	down
	Valine, leucine and isoleucine degradation	Ketol-acid reductoisomerase, mitochondrial	up
	Tyrosine metabolism	Probable 4-hydroxyphenylpyruvate dioxygenase 1	down
	Arginine and proline meta	Ornithine aminotransferase	down
	Phenylalanine, tyrosine, and tryptophan biosynthesis	Quinate dehydrogenase	down
Lipid metabolism	Fatty acid degradation	cytochrome P450 oxidoreductase	up
	Fatty acid biosynthesis	Fatty acid synthase subunit alpha	up
	Sphingolipid metabolism	Serine palmitoyltransferase 2	up
	Steroid biosynthesis	Eburicol 14-alpha-demethylase	up
		Sterol 24-C-methyltransferase	up
		C-5 sterol desaturase	up
		Methylsterol monooxygenase	up
	Biosynthesis of unsaturated fatty acids	Acyl-CoA desaturase	up
		Oleate hydroxylase FAH12	up

## Data Availability

Date will be made available on request.

## Supplementary Materials

The following supporting information can be downloaded at: https://www.mdpi.com/article/10.3390/jof9121174/s1, Figure S1: Gel electrophoresis map of construction and verification of RNA interference vectors and Monascus transformants. (a) Verification of the pC3300-*neo* plasmid using double-enzyme digestion. Lane 1 indicates the double-enzyme digestion product of the pC3300-*neo* plasmid; lane 2 indicates the PCR product of *neo* gene (the expected size is 1221 bp, primers are *Xho* I-*neo* Forward/ *Eco*RI-*neo* Reverse). (b) Obtaining the *PtrpC*-ihpRNA *pksCT*α-*TtrpC* fragment using Double-Joint PCR. Lanes 1 and 2 indicate the PCR products of *PtrpC* (α) and *TtrpC* (α) fragments, respectively (the expected size of *PtrpC* (α) is 395 bp, primers are *Hin*d III-*PtrpC* (α) Forward/ *PtrpC* (α)*-*Reverse; the expected size of *TtrpC* (α) is 630 bp, primers are *TtrpC* (α) Forward/ *Xba* I-*TtrpC* (α) Reverse). lane 3 indicates the Double-Joint PCR product of the *PtrpC*-ihpRNA *pksCT*α-*TtrpC* fragment (the expected size is 1025 bp, primers are *Hin*d III-*PtrpC* (α) Forward/ *Xba* I-*TtrpC* (α) Reverse). (c) PCR verification of the pC3300-*neo*- *PtrpC*-ihpRNA *pksCTα*-*TtrpC* plasmid. Lanes 1 and 2 indicate the PCR product of the *PtrpC*-ihpRNA *pksCT*α-*TtrpC* fragment and the *neo* gene (the expected size of *PtrpC*-ihpRNA *pksCT*α-*TtrpC* fragment is 1025 bp, primers are *Hin*d III-*PtrpC* (α) Forward/ *PtrpC* (α)*-*Reverse; t the expected size of *neo* is 1221 bp, primers are *Xho* I-*neo* Forward/ *Eco*RI-*neo* Reverse). (d) Obtaining the *PtrpC*-ihpRNA *pksCT* (α + β)-*TtrpC* fragment using Double-Joint PCR. Lanes 1 and 2 indicate the PCR product of *PtrpC* (α + β) and *TtrpC* (α + β) fragments (the expected size of *PtrpC* (α + β) is 395 bp, primers are *Hin*d III-*PtrpC* Forward/ *PtrpC-*Reverse; the expected size of *TtrpC* (α + β) is 630 bp, primers are *TtrpC* Forward/ *Xba* I-*TtrpC* Reverse); lane 3 indicates the double-joint PCR product of *PtrpC*-ihpRNA *pksCT* (α + β)-*TtrpC* fragment (the expected size is 1025 bp, primers are *Hin*d III-*PtrpC* Forward/ *Xba* I-*TtrpC* Reverse). (e) PCR verification of the pC3300-*neo*- *PtrpC*-ihpRNA *pksCT* (α + β)-*TtrpC* plasmid. Lanes 1 and 2 indicate the PCR product of the *PtrpC*-ihpRNA *pksCT* (α + β)-*TtrpC* fragment and the *neo* gene (the expected size of *PtrpC*-ihpRNA *pksCT* (α + β)-*TtrpC* is 1025 bp, primers are *Hin*d III-*PtrpC* Forward/*Xba* I-*TtrpC*; the expected of *neo* size is 1221 bp, primers are *Xho* I-*neo* Forward/ *Eco*RI-*neo* Reverse). (f) Confirmation of plasmid integration in *Monascus* transformants silencing *pksCT*α. Lanes 1 and 2 indicate the PCR product of the *PtrpC*-ihpRNA *pksCT*α-*TtrpC* fragment, with the genomic DNA and the recombinant plasmid as templates, respectively (the expected size is 1025 bp, primers are *Hin*d III-*PtrpC* (α) Forward/ *Xba* I-*TtrpC* (α) Reverse). (g) Plasmid integration confirmation of *Monascus* transformants silencing *pksCT* (α + β). Lanes 1 and 2 indicate the PCR product of the *PtrpC*-ihpRNA *pksCT* (α + β)-*TtrpC* fragment, with the genomic DNA and the recombinant plasmid as templates, respectively (the expected size is 1025 bp, primers are *Hin*d III-*PtrpC* Forward/*Xba* I-*TtrpC*); Figure S2: Length distribution of transcripts (A) and classification of new transcripts (B). e: single exon transfrag overlapping a reference exon and at least 10 bp of a reference intron, indication of a possible pre-mRNA fragment; i: transfrag falling entirely within a reference intron; j: potentially novel isoform (fragment): at least one splice junction is shared with a reference transcript; o: generic exonic overlap with a reference transcript; p: possible polymerase run-on fragment (within 2K bases of a reference transcript); s: An intron of the transfrag overlaps a reference intron on the opposite strand (likely due to read mapping errors); u: unknown, intergenic transcript; x: exonic overlap with reference on the opposite strand; =: complete match of the intron chain.; Figure S3: The differentially expressed genes (DEGs) with up and down regulation in three comparison groups (A) and the shared DEGs in the comparison groups between M7_vs_A and M7_vs_B (B). The blue ball represents the comparison group M7_vs_ A; red ball represents the comparison group M7_vs_B; M7: the wild-type strain, control group; group A: *Monascus* transformant with silenced *pksCT*α gene, the experimental group; group B: *Monascus* transformant with silenced *pksCT* (α + β) gene, the experimental group; Figure S4: Kyoto Encyclopedia of Genes and Genomes (KEGG) database enrichment analysis of the up-regulated (A) and down-regulated (B) differentially expressed genes in the comparison group A_vs_B. group A: *Monascus* transformant with silenced *pksCT*α gene, the experimental group; group B: *Monascus* transformant with silenced *pksCT* (α + β) gene, the experimental group; Figure S5: Kyoto Encyclopedia of Genes and Genomes (KEGG) database analysis of alternative splicing-related genes in the three comparison groups of M7_vs_A (A), M7_vs_B (B) and A_vs_B (C). M7: the wild-type strain, control group; group A: *Monascus* transformant with silenced *pksCT*α gene, the experimental group; group B: *Monascus* transformant with silenced *pksCT* (α + β) gene, the experimental group; Figure S6: Kyoto Encyclopedia of Genes and Genomes (KEGG) database analysis of up-regulated (A) and down-regulated (B) differentially expressed genes in the first comparison group M7_vs_A. M7: the wild-type strain, control group; group A: *Monascus* transformant with silenced *pksCT*α gene, the experimental group; Table S1: The primers used in RT-qPCR verification; Table S2: Reads quality of different *Monascus* samples; Table S3: Reads mapping with the reference genome.

## Abbreviations

CIT, citrinin; AS, alternative splicing; acetyl-coA, acetyl-coenzyme-A; malonyl-coA, malonyl-coenzyme-A; PDA, potato dextrose agar; YES, yeast extract sucrose; LB, Luria–Bertani; ihpRNA, hairpin RNA; ATMT, *Agrobacterium tumefaciens*-mediated transformation; DEGs, differentially expressed genes; FD, fold change; GO, gene ontology; KEGG, kyoto encyclopedia of genes and genomes; RT-qPCR, reverse transcription quantitative PCR; SCC, spearman’s correlation coefficient; COG, cluster of orthologous groups; MF, molecular function; BP, biological process; CC, cellular composition; TCA cycle, citrate cycle.
