# Supplementary material for: Metabolic Regulation of Two pksCT Gene Transcripts in Monascus ruber Impacts Citrinin Biosynthesis
Source: J Fungi (Basel). 2023 Dec 7;9(12):1174. doi: 10.3390/jof9121174 (PMC10745002; doi:10.3390/jof9121174)
Supplement: Supplementary file 1 [file jof-09-01174-s001.zip › jof-2699604-supplementary.pdf]

Supplementary Material

Metabolic Regulation of Two *pksCT* Gene Transcripts in *Monascus ruber* Impacts Citrinin Biosynthesis

Yi He [1,2,*,†], Lisha Zhu [1,2,†], Xingxing Dong [1], Aoran Li [2], Suyin Xu [2], Liling Wang [3] and Yanchun Shao [4,*]

[1] National R&D Center for Se-rich Agricultural Products Processing, Hubei Engineering Research Center for Deep Processing of Green Se-rich Agricultural Products, School of Modern Industry for Selenium Science and Engineering, Wuhan Polytechnic University, Wuhan 430023, China; zhulisha199888@126.com (L.Z.); dongxingxing@163.com (X.D.)
[2] Key Laboratory for Deep Processing of Major Grain and Oil, Ministry of Education, Hubei Key Laboratory for Processing and Transformation of Agricultural Products, School of Food Science and Engineering, Wuhan Polytechnic University, Wuhan 430023, China; liaoran618@outlook.com (A.L.); xqw1koss@163.com (S.X.)
[3] College of Food Science and Engineering, Tarim University, Alar 843300, China; 120060036@taru.edu.cn
[4] College of Food Science and Technology, Huazhong Agricultural University, Wuhan 430070, China
* Correspondence: yi.he@whpu.edu.cn (Y.H.); yanchunshao@mail.hzau.edu.cn (Y.S.); Tel.: +86-27-13419552316 (Y.H.); +86-27-18571701976 (Y.S.)
† The authors contributed equally to this work.

Supplementary Methods

Construction of the fungal expression vector pC3300-*neo*

Amplification of neomycin resistant gene was performed using a Bio-Rad T100 PCR amplifier (Bio-Rad, Hercules, CA, USA) with the following program: 94 °C for 3 min, followed by a three-step PCR (30 cycles of denaturation at 98 °C for 10 s, annealing at 55 °C for 15 s, and extension at 68 °C for 1 min). The PCR products of the *neo* and pC3300 plasmids were double-digested with QuickCut *Xho* I and QuickCut *Eco*R I and linked together using T4 DNA ligase after purification. The linking products were transformed into DH5α competent cells, and the positive transformants were screened on the selected medium, which contained 50 μg/mL kanamycin. The recombinant plasmid of the positive transformant was extracted and verified by electrophoresis, double-digestion and sequencing. ApexHFHS DNA Polymerase FL was obtained from Accurate Biotechnology

(Hunan) Co., Ltd. (Shanghai, China). QuickCut restriction enzymes and T4 DNA ligase were purchased from Takara Biomedical Technology (Beijing) Co., Ltd. (Beijing, China).

Plasmid construction for silencing *pksCTα* and *pksCT* (α + β) genes

1. Plasmid construction for silencing the *pksCTα* gene

The promoter *trpC* (*PtrpC*) and terminator *trpC* (*TtrpC*) with restriction sites were amplified by PCR from the pSKH plasmid using specific primers (Table 1) with the following program: 94 °C for 3 min, followed by a three-step PCR (30 cycles of denaturation at 98 °C for 10 s, annealing at 57.5 °C/67 °C (*PtrpC* (*α*)/*TtrpC* (*α*)) for 15 s, and extension at 68 °C for 1 min). After purification, *PtrpC* (*α*) and *TtrpC* (*α*) were connected by Double-Joint PCR using the primers *Hin*d Ⅲ-*PtrpC* Forward and *Xba* Ⅰ-*TtrpC* Reverse with the following program: 94 °C for 3 min, followed by a three-step PCR (35 cycles of denaturation at 98 °C for 10 s, annealing at 60 °C for 15 s, and extension at 68 °C for 90 s). The pC3300-*neo* vector and the joined product of *PtrpC*-ihpRNA *pksCTα*-*TtrpC* were double-digested with QuickCut *Hin*d III and QuickCut *Xba* I and linked together using T4 DNA ligase after purification. The linking products were transformed into DH5*α* competent cells, and then the recombinant plasmids of the positive transformants were extracted and verified.

2. Plasmid construction for silencing the *pksCT* (α + β) gene

PtrpC and *TtrpC* fragments containing restriction sites were amplified by PCR from the pSKH plasmid using specific primers (Table 1) with the following program: 94 °C for 3 min, followed by a three-step PCR (30 cycles of denaturation at 98 °C for 10 s, annealing at 56 °C (*PtrpC* (*α*+β)/*TtrpC* (*α*+β)) for 15 s, and extension at 68 °C for 1 min). After purification, *PtrpC* (*α*+β) and *TtrpC* (*α*+β) were connected by Double-Joint PCR using the primers *Hin*d Ⅲ-*PtrpC* Forward and *Xba* Ⅰ-*TtrpC* Reverse with the following program: 94 °C for 3 min, followed by a three-step PCR (35 cycles of denaturation at 98 °C for 10 s, annealing at 55 °C for 15 s, and extension at 68 °C for 90 s). The pC3300-*neo* vector and the joined product of *PtrpC*-ihpRNA *pksCT* (*α* + β)-*TtrpC* were double-digested with QuickCut *Hin*d III and QuickCut *Xba* I and linked together using T4 DNA ligase after purification.

The linking products were transformed into DH5α competent cells, and then the recombinant plasmids of the positive transformants were extracted and verified.

Confirmation of recombinant plasmids

1. Confirmation of pC3300-*neo-PtrpC*-ihpRNA-*pksCTα-TtrpC*

PtrpC (α) with 395 bp (lane 1) and *TtrpC* (α) with 630 bp (lane 2) were successfully amplified. Notably, the DNA band in lane 3 represented the combined size of *PtrpC* (α) and *TtrpC* (α), indicating successful fusion into the *PtrpC*-ihpRNA-*pksCTα-TtrpC* fragment (1025 bp) (Fig. S1B). After transforming the linking products of *PtrpC*-ihpRNA-*pksCTα-TtrpC* and pC3300-neo into DH5α-competent cells, PCR and gene sequencing confirmed the presence of both the *PtrpC*-ihpRNA-*pksCTα-TtrpC* fragment (lane 1) and the neo gene (lane 2) in the recombinant plasmid (Fig. S1C).

2. Confirmation of pC3300-*neo-PtrpC*-ihpRNA-*pksCT* (α + β)-*TtrpC*

Similar to constructing the recombinant plasmid for *pksCTα* gene silencing, the 395 bp *PtrpC* (α + β) fragment (lane 1) and the 630 bp *TtrpC* (α + β) fragment (lane 2) were amplified. Subsequently, these fragments were successfully fused to form the *PtrpC*-ihpRNA-*pksCT* (α + β)-*TtrpC* fragment with 1025 bp (lane 3) (Fig. S1D). Following transformation of the linking products of *PtrpC*-ihpRNA-*pksCT* (α + β)-*TtrpC* and pC3300-*neo* into DH5α competent cells, verification confirmed the integration of both the *PtrpC*-ihpRNA-*pksCT* (α + β)-*TtrpC* fragment (lane 1) and the *neo* gene (lane 2) into the pC3300 vector (Fig. S1E).

Plasmid integration confirmation

1. Confirmation of *Monascus* transformants with silenced *pksCTα*

The recombinant plasmid of pC3300-*neo-PtrpC*-ihpRNA *pksCTα-TtrpC* was successfully transformed into the parental strain M7 using the ATMT method, with subsequent screening for positive *Monascus* transformants. Genomic DNA analysis confirmed the integration of the ihpRNA-*pksCTα* fragment (lane 2) with the ihpRNA-*pksCTα* transformant, matching the size of the PCR product (lane 1) amplified from the pC3300-*neo-PtrpC*-ihpRNA-*pksCTα-TtrpC* plasmid (Fig. S1F).

2. Confirmation of *Monascus* transformants with silenced *pksCT* (α + β)

Fig. S1G shows the successful integration of the ihpRNA-*pksCT* (α + β) fragment (lane 2) with the genomic DNA of the ihpRNA-*pksCT* (α + β) transformant, which was consistent in size with the PCR product (lane 1) amplified from the pC3300-*neo*-*PtrpC*-ihpRNA-*pksCT* (α + β)-*TtrpC* plasmid. Notably, no remarkable differences in growth rate or total MP production were observed between M7 and the *Monascus* transformants.

Measurement of CIT content

Pure CIT of HPLC grade was purchased from Sigma-Aldrich (Shanghai) Trading Co., Ltd. (Shanghai, China), and the concentration of CIT was calculated based on the peak area of pure CIT. Briefly, the fermentation broth was mixed with the same volume of toluene/ethylacetate/formic acid buffer (7:3:1, v:v:v) and centrifuged at 13,400 ×g at 4℃ for 10 min to collect the supernatant, which contained CIT. The supernatant was transferred into a beaker for evaporation drying at room temperature, then 80% methanol of HPLC grade was added to dissolve the dried CIT. After filtering through a 0.22 μm filter, the samples were analyzed using a fluorescence detector of the UPLC system (Waters) with a Waters C18 chromatographic column (1.7 μm, 2.1 mm×100 mm). The mobile phase solvent A was acetonitrile, and solvent B was 0.1% (v/v) formic acid in water. CIT was separated at a flow rate of 1.0 mL/min with a column temperature of 40 °C under the following conditions: 0-3 min, 90% A and 10% B; 3-10 min, 30% A and 70 % B; 10-12 min, 10% A and 90% B; 12-15 min, 90% A and 10% B. The excitation and emission wavelengths were 330 nm and 500 nm, respectively.

RT-qPCR analysis of genes involved in CIT biosynthesis

The *Monascus* samples were cultivated on YES medium at 28℃ and 150 rpm for 5 days, and the total RNA was extracted using AG RNAex Pro Reagent (Accurate Biotechnology (Hunan) Co., Ltd., Hunan, China). Only the high-quality RNA samples (OD260/280 = 1.8~2.2, OD260/230 ≥ 2.0, 28S: 18S ≥ 1.0) were used to reverse transcribe and quantify the samples. The reverse transcription was performed using the Evo M-MLV RT Kit with gDNA Clean for qPCR, while the quantification was done using the SYBR® Green Premix Pro TaqHS qPCR kit (Accurate Biotechnology (Hunan) Co., Ltd., Hunan, China), respectively. The relative expression level of each gene was normalized to glyceraldehyde-

3-phosphate dehydrogenase (GAPDH) and analyzed using the $2^{-\Delta\Delta CT}$ method. The primers used in RT-qPCR are listed in Supplementary Table S1.

Supplementary Figures

Figure S1 Gel electrophoresis map of construction and verification of RNA interference vectors and *Monascus* transformants. (A) Verification of the pC3300-*neo* plasmid using double-enzyme digestion. Lane 1 indicates the double-enzyme digestion product of the pC3300-*neo* plasmid; lane 2 indicates the PCR product of *neo* gene (the expected size is 1221 bp, primers are *Xho* Ⅰ-*neo* Forward/ *Eco*R Ⅰ-*neo* Reverse). (B) Obtaining the *PtrpC*-ihpRNA *pksCTα*-*TtrpC* fragment using Double-Joint PCR. Lanes 1 and 2 indicate the PCR products of *PtrpC* (*α*) and *TtrpC* (*α*) fragments, respectively (the expected size of *PtrpC* (*α*) is 395 bp, primers are *Hin*d Ⅲ-*PtrpC* (*α*) Forward/ *PtrpC* (*α*)-Reverse; the expected size of *TtrpC* (*α*) is 630 bp, primers are *TtrpC* (*α*) Forward/ *Xba* Ⅰ-*TtrpC* (*α*) Reverse). lane 3 indicates the Double-Joint PCR product of the *PtrpC*-ihpRNA *pksCTα*-*TtrpC* fragment (the expected size is 1025 bp, primers are *Hin*d Ⅲ-*PtrpC* (*α*) Forward/ *Xba* Ⅰ-*TtrpC* (*α*) Reverse). (C) PCR verification of the pC3300-*neo*- *PtrpC*-ihpRNA *pksCTα*-*TtrpC* plasmid. Lanes 1 and 2 indicate the PCR product of the *PtrpC*-ihpRNA *pksCTα*-*TtrpC* fragment and the *neo* gene (the expected size of *PtrpC*-ihpRNA *pksCTα*-*TtrpC* fragment is 1025 bp, primers are *Hin*d Ⅲ-*PtrpC* (*α*) Forward/ *PtrpC* (*α*)-Reverse; t the expected size of *neo* is 1221 bp, primers are *Xho* Ⅰ-*neo* Forward/ *Eco*R Ⅰ-*neo* Reverse). (D) Obtaining the *PtrpC*-ihpRNA *pksCT* (α + β)-*TtrpC* fragment using Double-Joint PCR. Lanes 1 and 2 indicate the PCR product of *PtrpC* (α + β) and *TtrpC* (α + β) fragments (the expected size of *PtrpC* (α + β) is 395 bp, primers are *Hin*d Ⅲ-*PtrpC* Forward/ *PtrpC*-Reverse; the expected size of *TtrpC* (α + β) is 630 bp, primers are *TtrpC* Forward/ *Xba* Ⅰ-*TtrpC* Reverse); lane 3 indicates the double-joint PCR product of *PtrpC*-ihpRNA *pksCT* (α + β)-*TtrpC* fragment (the expected size is 1025 bp, primers are *Hin*d Ⅲ-*PtrpC* Forward/ *Xba* Ⅰ-*TtrpC* Reverse). (E) PCR verification of the pC3300-*neo*-*PtrpC*-ihpRNA *pksCT* (α + β)-*TtrpC* plasmid. Lanes 1 and 2 indicate the PCR product of the *PtrpC*-ihpRNA *pksCT* (α + β)-*TtrpC* fragment and the *neo* gene (the expected size of *PtrpC*-ihpRNA *pksCT* (α + β)-*TtrpC* is 1025 bp, primers are *Hin*d Ⅲ-*PtrpC* Forward/*Xba* Ⅰ-*TtrpC*; the expected of *neo* size is 1221 bp, primers are *Xho* Ⅰ-*neo* Forward/ *Eco*R Ⅰ-*neo* Reverse). (F) Confirmation of plasmid integration in *Monascus* transformants silencing *pksCTα*. Lanes 1 and 2 indicate the PCR product of the *PtrpC*-ihpRNA *pksCTα*-*TtrpC* fragment, with

the genomic DNA and the recombinant plasmid as templates, respectively (the expected size is 1025 bp, primers are *Hin*d Ⅲ-*PtrpC* (*α*) Forward/ *Xba* Ⅰ-*TtrpC* (*α*) Reverse). (G) Plasmid integration confirmation of *Monascus* transformants silencing *pksCT* (*α* + β). Lanes 1 and 2 indicate the PCR product of the *PtrpC*-ihpRNA *pksCT* (*α* + β)-*TtrpC* fragment, with the genomic DNA and the recombinant plasmid as templates, respectively (the expected size is 1025 bp, primers are *Hin*d Ⅲ-*PtrpC* Forward/*Xba* Ⅰ-*TtrpC*).

Figure S2 Length distribution of transcripts (A) and classification of new transcripts (B). e: single exon transfrag overlapping a reference exon and at least 10 bp of a reference intron, indication of a possible pre-mRNA fragment; i: transfrag falling entirely within a reference intron; j: potentially novel isoform (fragment): at least one splice junction is shared with a reference transcript; o: generic exonic overlap with a reference transcript; p: possible polymerase run-on fragment (within 2K bases of a reference transcript); s: An intron of the transfrag overlaps a reference intron on the opposite strand (likely due to read mapping errors); u: unknown, intergenic transcript; x: exonic overlap with reference on the opposite strand; =: complete match of the intron chain.

Figure S3 The differentially expressed genes (DEGs) with up and down regulation in three comparison groups (A) and the shared DEGs in the comparison groups between M7_vs_A and M7_vs_B (B). The blue ball represents the comparison group M7_vs_ A; red ball represents the comparison group M7_vs_B.

M7: the wild-type strain, control group; group A: *Monascus* transformant with silenced *pksCTα* gene, the experimental group; group B: *Monascus* transformant with silenced *pksCT* (*α* + β) gene, the experimental group.

Figure S4 Kyoto Encyclopedia of Genes and Genomes (KEGG) database enrichment analysis of the up-regulated (A) and down-regulated (B) differentially expressed genes in the comparison group A_vs_B.

group A: *Monascus* transformant with silenced *pksCTα* gene, the experimental group; group B: *Monascus* transformant with silenced *pksCT* (*α* + β) gene, the experimental group.

Figure S5 Kyoto Encyclopedia of Genes and Genomes (KEGG) database analysis of alternative splicing-related genes in the three comparison groups of M7_vs_A (A), M7_vs_B (B) and A_vs_B (C).

M7: the wild-type strain, control group; group A: *Monascus* transformant with silenced *pksCTα* gene, the experimental group; group B: *Monascus* transformant with silenced *pksCT* (α + β) gene, the experimental group.

Figure S6 Kyoto Encyclopedia of Genes and Genomes (KEGG) database analysis of up-regulated (A) and down-regulated (B) differentially expressed genes in the first comparison group M7_vs_A.

M7: the wild-type strain, control group; group A: *Monascus* transformant with silenced *pksCTα* gene, the experimental group.

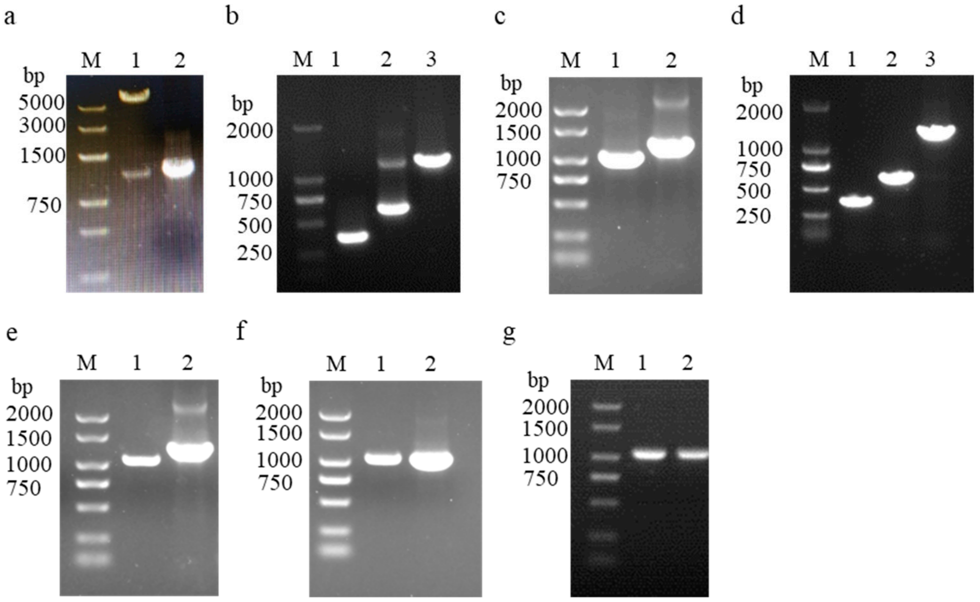

Figure S1

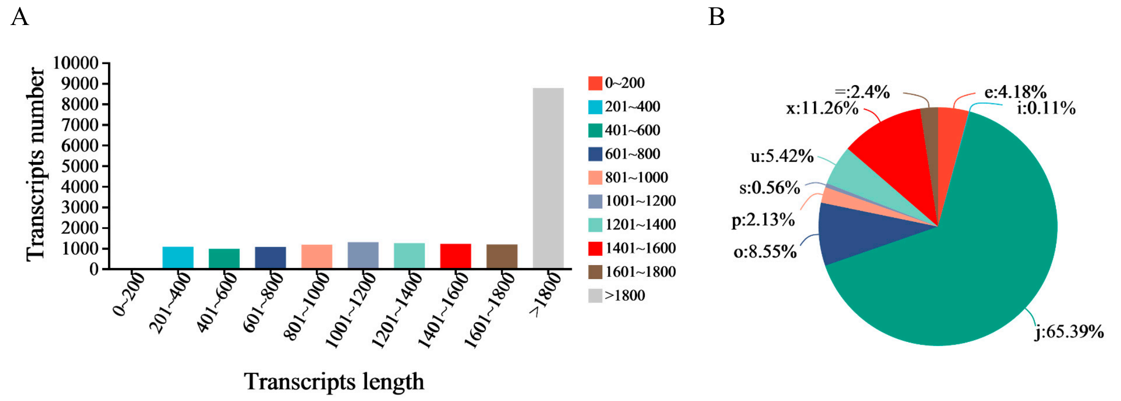

Figure S2

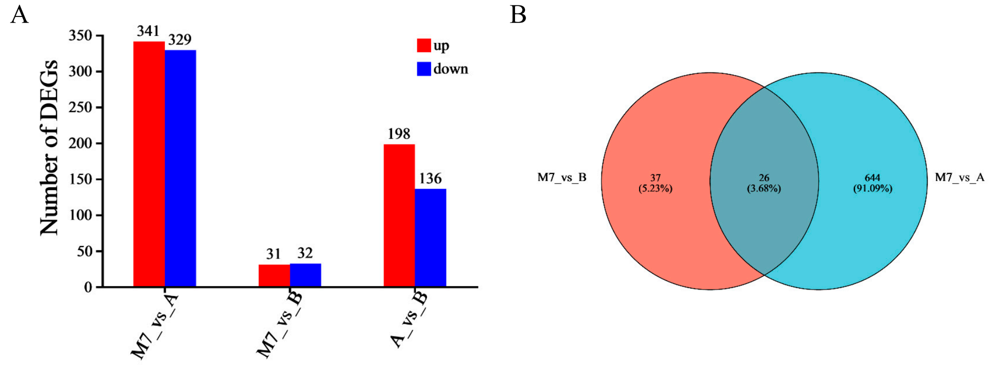

Figure S3

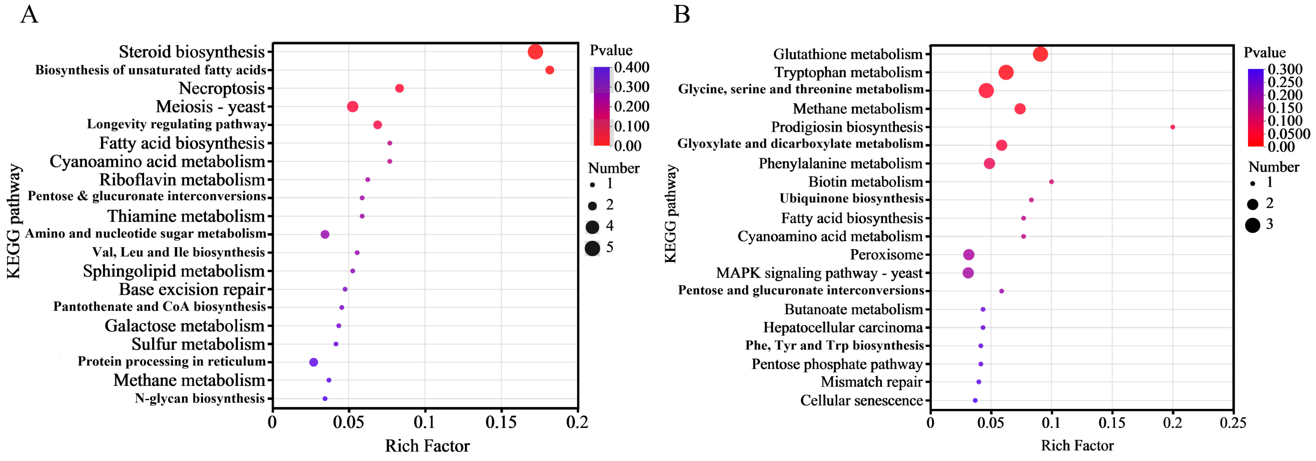

Figure S4

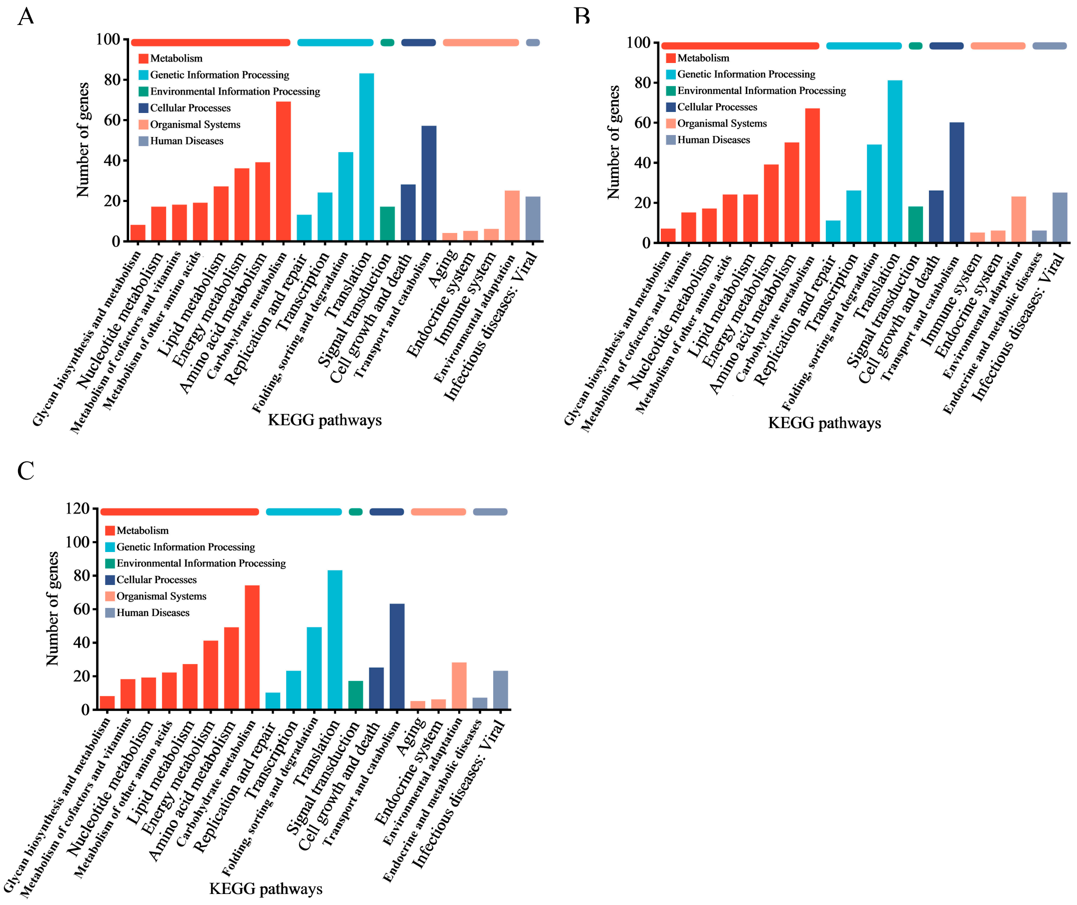

Figure S5

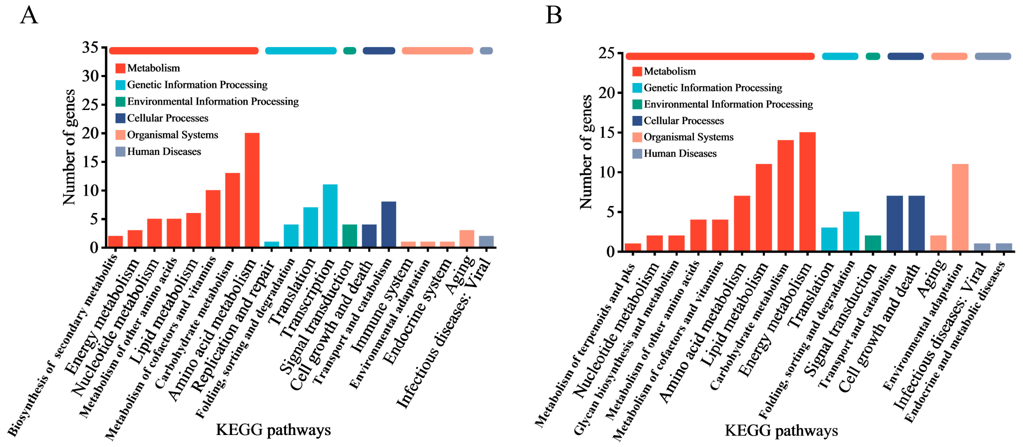

Figure S6

Supplementary Tables

Table S1 The primers used in RT-qPCR verification.

Table S2 Reads quality of different *Monascus* samples.

Table S3 Reads mapping with the reference genome.

Table S1

Name	Sequence (5′-3′)
GAPDH-F	CAAGCTCACTGGCATGTCTATG
GAPDH-R	AAGTTCGAGTTGAGGGCGATA
pksCT-F	AATCTGCGGTCATAGTCTCTTC
pksCT-R	CGAGGCTATGAGAGTCAGTTC
mrl5-F	GATCTGCATTCTACAAGGAGG
mrl5-R	TTCGAAGGTTTGAAGCTTGTG
mrr1-F	TGGATGTCCCTCTGGGTGA
mrr1-R	GGTCGAGACGTGGAACTT
mrr5-F	GCATCACGCTATATAGTT
mrr5-R	CTCATACATTGCACATTG

Sample	Raw reads	Raw bases	Clean bases	Clean reads	Error rate (%)	Q20 (%)	Q30 (%)	GC content (%)
M_2_1	42654994	6.44E+09	42160818	6.14E+09	0.0244	98.23	94.72	53.25
M_3_1	43238170	6.53E+09	42649204	6.2E+09	0.0249	98.05	94.31	53.18
M_4_1	44661786	6.74E+09	44026620	6.43E+09	0.0258	97.66	93.39	52.87
a_3_1	45898386	6.93E+09	45168080	6.67E+09	0.024	98.45	95.2	53.39
a_6_1	47795118	7.22E+09	47280686	6.89E+09	0.0247	98.11	94.44	52.31
a_8_1	46080384	6.96E+09	45546978	6.64E+09	0.0248	98.07	94.31	52.74
b_1_1	42244346	6.38E+09	41707918	6.14E+09	0.0256	97.76	93.61	53.16
b_7_1	52695506	7.96E+09	52112776	7.57E+09	0.0249	98.04	94.3	53.19
b_8_1	45639736	6.89E+09	45072906	6.61E+09	0.025	97.97	94.17	52.73

Table S2

Sample	Total reads	Total mapped	Multiple mapped	Uniquely mapped
M_2_1	42160818	39903863 (94.65%)	189043 (0.45%)	39714820 (94.2%)
M_3_1	42649204	40417507 (94.77%)	202059 (0.47%)	40215448 (94.29%)
M_4_1	44026620	41735740 (94.8%)	201397 (0.46%)	41534343 (94.34%)
a_3_1	45168080	39529183 (87.52%)	187765 (0.42%)	39341418 (87.1%)
a_6_1	47280686	44392321 (93.89%)	162096 (0.34%)	44230225 (93.55%)
a_8_1	45546978	43272667 (95.01%)	147463 (0.32%)	43125204 (94.68%)
b_1_1	41707918	39095439 (93.74%)	160831 (0.39%)	38934608 (93.35%)
b_7_1	52112776	48665872 (93.39%)	238048 (0.46%)	48427824 (92.93%)

b_8_1	45072906	42738120 (94.82%)	184670 (0.41%)	42553450 (94.41%)

Table S3
